# Experiences of victimization, maladaptive schemas, and the mediating role of resilience in adolescents

**DOI:** 10.1186/s40359-026-04275-1

**Published:** 2026-04-09

**Authors:** Vanessa Caba-Machado, Raquel Escortell, Joaquín González-Cabrera, Eider González-Abaurrea, Adoración Diáz-López, Juan M. Machimbarrena

**Affiliations:** 1 Instituto de Transferencia e Investigación (ITEI), Universidad Internacional de La Rioja (UNIR), Logroño, La Rioja Spain; 2 Facultad de Psicología, Universidad del País Vasco (UPV/EHU), Avda. Tolosa Hirib, 70 20018 Donostia - San Sebastián, Spain; 3 Facultad de Educación. Universidad Internacional de La Rioja (UNIR), Logroño, Spain

**Keywords:** Victimization, Cybervictimization, Resilience, Maladaptive schemas, Adolescence

## Abstract

**Background:**

Numerous studies have established the relationship between peer victimization and maladaptive schemas. Some studies indicate that resilience mediates between victimization and psychological distress, although there is a gap in the literature on the role of resilience in the formation of maladaptive schemas.

**Aim:**

This study aimed to analyze the role of resilience as a mediator between peer victimization, cybervictimization, and maladaptive schemas, especially disconnection/rejection and impaired autonomy.

**Methods:**

An analytical and cross-sectional study was performed with 2854 students (50.7% girls) aged 11–17 years (Mage = 13.71, SD = 1.34) from 16 schools in six Spanish regions. Confirmatory factor analyses (CFA) and structural equation models (SEM) were performed, and multigroup measurement invariance by sex was confirmed.

**Results:**

The results show that resilience in all its dimensions acts as a partial mediator between traditional victimization and maladaptive schemas, particularly in the dimensions of self-efficacy and control under pressure. However, resilience only shows a partial mediation in cybervictimization through the dimension of Spirituality. Moreover, the model is invariant for boys and girls.

**Conclusion:**

Resilience dimensions partially mediate the victimization–schema association, cybervictimization shows weaker mediation effects, and the model is invariant across sex.

## Introduction

Interpersonal violence is a fact that has currently become a common practice worldwide [1] and, therefore, a public health problem [2]. In turn, victimization—an essential part of this dynamic—is a phenomenon of profound social relevance with a negative impact on the well-being of those affected in the short and long term [3]. This phenomenon also applies to the online context, with cybervictimization being described as a contemporary variant whose prevalence has increased significantly due to the rise and consolidation of Relational, Information and Communication Technologies (RICTs) in the different stages of childhood and adolescence [4, 5]. The online nature of cybervictimization adds complexity to the problem, as victims may face aggression without a clearly defined safe space, exacerbating the difficulty of addressing and preventing this type of harmful behavior [6]. Both dynamics give rise to the proliferation of a hostile environment that not only affects the emotional and psychological well-being of the individual in the present but can also have repercussions throughout their development [7]. In addition, there is a connection between these concepts, with co-occurrence or bidirectionality between them [8, 9]. This is probably because, based on the theory of the co-construction of reality [10], adolescents' problems in the school context continue in the digital world [11].

This combined version (face to face and online), which unifies both phenomena under the label of "victimization," are associated with lower levels of rejection and impaired autonomy schemas in the context of victimization [12] and is increasingly present in the lives of young people. In this regard, internationally, one in ten children claims to have been a victim of bullying [13], whereas between 14 and 57.5% have been cybervictims [13, 14]. In the Spanish context, the UNICEF report indicates a prevalence of peer victimization in the Compulsory Secondary Education (CSE) stage that varies between 19.9 and 33.6%, whereas, in the case of cybervictimization, it varies between 12.2 and 22.5% [15]. When sex differences are considered, there are no clear conclusions. Some studies have indicated that boys perform more violent behaviors and suffer more victimization and more directly than girls [16–19]. On the other hand, other studies [20–22] suggest that there is a greater tendency for girls to be victims and a greater tendency for boys to be bullies. It is likely that these differences are mainly due to the type of behavior performed (verbal, social, physical, psychological, etc.), whether it is direct or indirect, and the stage of adolescence [23, 24].

In general, the processes of peer violence have shown an impact on clinical variables and bio-psycho-social functioning [25]. Specifically, victimization has been associated with internalizing, externalizing, behavioral, social, and academic problems [26–29], poorer quality of life [23], and even with self-injurious behaviors and suicidal ideation [30–32], and the symptoms are more pronounced in stable victims over time [33]. Therefore, it is evident that physical and online victimization not only generates unpleasant external experiences but also influences the internal universe of the victims, interfering with the construction of their thoughts, emotions, and attitudes [34]. All of this can affect the victims' fundamental beliefs about themselves and the world around them, and can be a driving force in the consolidation of maladaptive schemas [35, 36].

The schema therapy model, developed by Young et al. [37], has been extensively studied to understand the impact of traumatic experiences on mental health. They have been defined as "broad and generalized patterns, linked to memories, emotions, cognitions, and bodily sensations, with respect to oneself and relationships with others, developed during childhood or adolescence, elaborated throughout life, and dysfunctional to a significant degree” [37]. This model proposes that negative experiences such as abuse or mistreatment contribute to the development of various psychological problems [38]. Maladaptive schemas are originated, in many cases, from early traumatic experiences, such as victimization, which negatively affect central self-evaluations in adolescence, youth, or adulthood [35, 36, 39].

Scientific literature has consistently demonstrated the relationship between victimization (face to face and online) and impaired cognitive, emotional, and relational functioning [25]. In particular, bullying [40] and cyberbullying [41] have been shown to predict a worsening of cognitive schemas. Furthermore, in-person or electronic victimization has been associated with greater impairment of body image and higher levels of interpersonal mistrust [42]. These findings highlight that victimization not only generates negative external experiences but also interferes with the construction of victims' internal world, influencing their thoughts, emotions, and attitudes [34], and potentially consolidating stable maladaptive schemas over time.

Similarly, maladaptive schemas are not only affected by interpersonal victimization but can also contribute to its perpetuation over time. In fact, it has been noted that maladaptive schemas maintain victimization continuously [43] and increase the risk of revictimization [35, 36]. Furthermore, it has been shown that these schemas can be transmitted across generations through adverse childhood experiences, especially when there is low parental support [44], and that they generate a negative predisposition toward present and future social interactions [45].

Within the model proposed by Young et al. [37], maladaptive schemas are organized into different domains. Among these, the disengagement/rejection domain has been identified as one of the most relevant in the context of interpersonal victimization. This domain includes schemas characterized by the expectation that basic needs for safety, acceptance, affection, and respect will not be met in a predictable way. Empirical evidence has shown that disengagement/rejection schemas can result from victimization experiences in different contexts, such as the family [46], school [40, 42], and digital environments [25, 38, 41, 47]. Furthermore, it has been observed that girls tend to score higher than boys on maladaptive schemas linked to rejection [48]. Given that victimization involves a repeated disruption of experiences of belonging, support, and social validation, the analysis of this domain is especially relevant to understanding its psychological consequences, as well as whether its effect can be moderated by personal adjustment variables.

On the other hand, there is the domain of impaired autonomy, which is the most controversial in terms of prior evidence. According to the original conceptualization by Young et al. [37] and its subsequent adaptations [49, 50], it is related to beliefs of failure, thoughts of inability to manage daily responsibilities, exaggerated fear of catastrophe, and excessive emotional involvement. Analysis of this domain has generated contradictory empirical results in the literature. While some studies have indicated that impaired autonomy is not as relevant in the context of victimization by school bullying or cyberbullying [35, 36], others have reported that this domain is indeed affected in situations of interpersonal violence [51]. Furthermore, impaired autonomy has been shown to be a particularly relevant domain for the development of depression [42, 48, 52] and other psychological symptoms [53]. This lack of empirical consensus highlights the need for further research into this domain in relation to experiences of victimization.

In any case, maladaptive schemas maintain interpersonal victimization on a continuous basis [43] and increase the risks of re-victimization [35, 36]. This reality points to the need to create work models focused on the victims's internal world, developing cognitive and emotional strategies for their management and overcoming. Precisely, the contributions of Positive Psychology are one of the current frameworks with the strongest tendency to develop strategies for managing and overcoming traumatic situations.

Positive Psychology emerged at the end of the twentieth century, and its purpose is to promote well-being, which requires positive social interactions, establishing goals, and achieving satisfaction [54]. Linked to the concept of well-being in Positive Psychology, resilience is a key concept [55]. Resilience is defined as the ability to adapt to adversity, highlighting the capacity to face challenges, overcome difficult situations, and recover from negative experiences [56]. In this study, resilience is conceptualized as a dynamic psychological resource that can be shaped by adverse interpersonal experiences and that, in turn, may influence cognitive and emotional adjustment processes, rather than as a fixed personality trait [57]. Given its function, it has been widely related to victimization [58–61], and is a great ally as a protective factor for victims [56, 62–64]. Likewise, the mediating nature of resilience between victimization and depressive moods [65, 66], life satisfaction [67], internet addiction [68], as well as between early experiences of rejection and bully victimization has been analyzed [47]. Therefore, it is clear that resilience is a psychological resource that helps people to react, face, and persist in the face of obstacles, such as bullying and cyberbullying, and that can be influenced by adverse interpersonal experiences. In addition, more specifically, it has been shown that the most relevant factors in this relationship are self-efficacy, religiosity/spirituality, social commitment, and culture [69].

Regarding gender differences, previous research has consistently shown that girls tend to report higher levels of traditional bullying and cybervictimization and are more likely to experience internalizing problems, whereas boys more often exhibit externalizing behaviours (e.g., aggression or rule-breaking). At the same time, several studies suggest that resilience plays a protective role for both sexes in the context of bullying and cyberbullying, with broadly similar associations between resilience and indicators of adjustment among victimized boys and girls [56, 70, 71]. However, to our knowledge, very few studies have explicitly examined whether a comprehensive mediation model linking peer victimization and cybervictimization, resilience, and maladaptive schemas is equivalent across boys and girls. This gap makes it difficult to determine whether the same explanatory model can be applied to both sexes or whether gender-specific models are needed, and it limits the development of evidence-based, resilience-focused interventions.

## Present study

The previous points have confirmed that the relationship between victimization and the formation of maladaptive schemas is well-documented in the scientific literature. Likewise, the role of resilience in victims' coping strategies and protection against bullies has also been confirmed [56, 62, 64, 72]. However, there is no conclusive evidence about the possible mediating role of resilience in the relationship between victimization experiences and the formation of maladaptive schemas. Broadly speaking, [39] established that resilience mediates the relationship between victimization and negative cognitive bias. Similarly, resilience has been shown to moderate the relationship between victimization and negative outcomes in terms of psychosocial adaptation and subjective well-being [73–75], and to protect against the negative factors of victimization [56]. Findings along the same lines affirm that resilience also partially mediates the relationship between emotional schemas and psychological distress [76]. However, there is still a gap in the scientific literature that needs to be specifically analyzed regarding the function of resilience between victimization and the formation of schemas. This is crucialfor developing possible cognitive and emotional strategies for victims' management and recovery, because resilience is a variable that can be addressed within the framework of prevention programs or from clinical care. It should be noted that, given the cross-sectional design of the study, the proposed mediation model represents a theoretically informed explanatory framework rather than evidence of causal ordering between victimization, resilience, and maladaptive schemas.

Therefore, the main objective of this study is to analyze the role of resilience as a mediator between peer victimization and cybervictimization and maladaptive schemas, especially those of disconnection/rejection and impaired autonomy. Secondarily, we propose to examine whether the mediation model is invariant according to sex, evaluating the fit of the SEM model in boys and girls and its multigroup invariance. It should be noted that, given the cross-sectional design of the study, the proposed mediation model represents a theoretically informed explanatory framework rather than evidence of causal ordering between victimization, resilience, and maladaptive schemas.

In this study, in the absence of previous studies, the following research questions are raised:

- RQ1: Does resilience totally or partially mediate between peer victimization/cybervictimization and maladaptive schemas of disconnection/rejection and impaired autonomy?
- RQ2: Is the mediation model sex-invariant?

## Material and method

### Design and participants

The study design was analytical and cross-sectional. The initial sample comprised 2963 students. However, after an exhaustive screening of the database (eliminating cases with incorrect answers to attention questions, abnormally short response times, and extreme scores), the final sample comprised 2854 participants (50.7% girls; 47.2 boys; 2.1% preferred not to answer) aged between 11 and 17 years (*M*_*age*_ = 13.71, *SD* = 1.34). Of these, 681 (52% girls) correspond to 1 st grade of Compulsory Secondary Education (CSE), 686 (53.8% girls) to 2nd grade of CSE, 666 (44.9% girls) to 3rd grade CSE, 589 (52% girls) to 4th grade of CSE, and 232 (51.3% girls) to 1 st year of high school. Incidental nonprobabilistic sampling was carried out. These samples belong to 16 schools financed with public funds in six Spanish regions (Andalusia, Catalonia, Aragon, Castilla y León, La Rioja, and the Basque Country). All the schools were located in urban areas.

### Assessment instruments

For the evaluation process, the following sociodemographic data were obtained: sex, age, grade, and school. The following assessment instruments were applied:

Cybervictimization dimension of the Cyberbullying Triangulation Questionnaire (CTQ) [77, 78]. Only the adapted dimension of cybervictimization was used. This consists of 9 items that reflect the most common behaviors in cyberbullying (sending threatening messages or humiliating images, exclusion from groups, etc.). The responses to this Likert scale range from 0 (*never*) to 4 (*almost every week*), so the total score can vary between 0 and 36 points. Participants were asked about experiences during the previous four months. In the present study, this dimension showed adequate internal consistency (α = 0.76; ω = 0.78).

Victimization dimension of the Spanish version of the European Bullying Intervention Project Questionnaire (EBIPQ) [79]. It presents 7 items that collect information on direct or indirect physical behaviors (e.g., hitting, pushing, or breaking things), verbal behaviors (e.g., related to insults, nicknames, shouting, etc.), social behaviors (e.g., isolation and harm to social relationships), and, finally, psychological behaviors (e.g., threats). They are rated on a 5-point Likert scale (0 = *never,* 4 = *always*). The total score ranges between 0 and 28 points. We asked about the last four months. Reliability indices indicated good internal consistency (α = 0.81; ω = 0.81).

Two dimensions of the Spanish version of the Young Schema Questionnaire-3 (YSQ-3) [48], Young 2018) were used to evaluate disconnection and rejection schemas, on the one hand and, on the other, impaired autonomy schemas. Thirty items were used for these two dimensions. The rejection schemas included items with content related to distrust, emotional deprivation, defectiveness, and abandonment (e.g., “*I have a feeling that people will take advantage of me"* or "*No one I desire would want to be close to me if they really knew me”*). Autonomy schemas included content related to vulnerability to harm, which implies an exaggerated fear that a catastrophe could occur at any time and that one will be unable to avoid it (e.g., “*I'm worried about being attacked")* and failure (e.g., “*I'm incompetent when it comes to achievements”*). Participants rated each item on a 6-point scale ranging from 1 (*completely false*) to 6 (*describes me perfectly*)*.* The disconnection and rejection dimension showed excellent reliability (α = 0.93; ω = 0.93), as did the impaired autonomy dimension (α = 0.90; ω = 0.90).

The Spanish version of the Connor-Davidson Resilience Scale (CD-RISC) for students was used to measure resilience [80]. The original scale in English consists of 25 items that form five factors [57]: self-efficacy-tenacity (8 items), control under pressure (7 items), adaptability and support networks (5 items), control and purpose (3 items), and spirituality (2 items). In addition to the aforementioned dimensions, two other structures have been proposed: a version that retains all 25 items loading on a second-order general factor model (CD-RISC25) and a shorter 10-item scale that loads on a single factor (CD-RISC10). Since this instrument was initially validated with undergraduate populations, we conducted CFA analyses of these three models, obtaining adequate fit indices in all cases: the five-dimensional model (χ^2^ = 2206.04, df = 265; RMSEA = 0.050; CFI = 0.924; SRMR = 0.035), the second-order model CD-RISC25 (χ^2^ = 2284.99, df = 270; RMSEA = 0.051; CFI = 0.921; SRMR = 0.035), and the CD-RISC10 (χ^2^ = 252.68, df = 35; RMSEA = 0.046; CFI = 0.974; SRMR = 0.023), with the latter showing the best overall fit. The alpha coefficient for the CD-RISC25 was 0.94, and for the CD-RISC10, it was 0.89. Regarding the five dimensions, reliability indices ranged from acceptable to good: self-efficacy–tenacity (α = 0.89; ω = 0.89), control under pressure (α = 0.81; ω = 0.81), adaptability and support networks (α = 0.74; ω = 0.74), control and purpose (α = 0.74; ω = 0.74), and spirituality, which showed lower reliability due to its brevity (α = 0.53; ω = 0.57). Despite its lower reliability, the spirituality dimension was retained in the analyses to preserve the theoretical integrity of the original CD-RISC model and to allow an exploratory examination of its potential differential role in victimization contexts, particularly in relation to cybervictimization.

### Ethical considerations

The study protocol was reviewed and approved by the Research Ethics Committees of Universidad del País Vasco and Universidad Internacional de La Rioja. All procedures complied with the principles of the Declaration of Helsinki and with Spanish and European regulations on personal data protection (EU General Data Protection Regulation 2016/679 and Organic Law 3/2018).

Prior to data collection, school directors received written information about the aims and procedures of the project and provided institutional permission for the study to be conducted in their schools. Caregivers or legal guardians were sent an information sheet and a written informed consent form. These documents explained the general objective of the study (to analyse adolescents’ experiences of peer victimization, cybervictimization, resilience and psychological adjustment), stated that participation was completely voluntary and that students could refuse or withdraw at any time without any academic or personal consequences, and specified that no economic incentives would be offered and that no foreseeable risks beyond the minimal discomfort associated with answering questions about everyday experiences were expected. The information sheet further specified that questionnaires were anonymous, that individual responses would be treated confidentially and used only for research purposes, and that results would be reported exclusively in aggregated form. Only students whose caregivers returned a signed informed consent form and who themselves agreed to participate completed the questionnaire (less than 1% of the caregivers or legal guardians of the minors did not allow their children’s participation).

On the day of the assessment, teachers followed a standardized script prepared by the research team to remind students of the purpose of the study, the approximate duration of the questionnaire, the voluntary nature of their participation, their right to skip any question they did not wish to answer or to stop participating at any time, and the confidential and anonymous treatment of their data. Students were also informed that neither teachers nor peers would have access to their individual answers and that any doubts could be addressed by the teacher or the school guidance department.

### Procedure

Participants completed the assessment through an online questionnaire platform. This process took place in computer classrooms and school classrooms through mobile devices. Classroom teachers and tutors participated in this process, coordinated by the guidance departments of each school. Before starting the questionnaire, teachers read a brief standardized script that reminded students of the aims of the study, the approximate duration of the assessment, the voluntary nature of their participation, and the anonymity and confidentiality of their responses, and explicitly informed them that they could stop answering at any moment without any negative consequences. All of them received specific instructions on the procedure to promote its standardization. The researchers monitored the online collection of data with the records of each school for all groups and classes. The time needed to complete the questionnaires ranged from 12 to 21 min, depending on the students' age and reading comprehension.

Mechanisms were enabled to detect problems in student responses [81]. Once the total sample of 2963 students was collected, the database was screened and filtered, eliminating the following cases: 1) those questionnaires with responses that were two standard deviations below the average response time of the sample,2) incomplete questionnaires in more than one item. The few missing values (< 5%) of the sample were imputed by the modal value of the item for their sample.

### Data analysis

The SPSS software (version 29; IBM Corp.) was used to: (1) explore and screen all data through descriptive statistics; (2) assess reliability using Cronbach’s α, ω coefficients, and normality through skewness and kurtosis; and (3) examine the relationships between variables using bivariate correlations. Absolute values of skewness and kurtosis were considered normal if skewness was below ± 3 and kurtosis below ± 10, based on Bentler and Bonett [82].

Additionally, AMOS software (version 29) was employed to: (1) conduct confirmatory factor analyses (CFA) and structural equation modeling (SEM); and (2) test multigroup invariance of the SEM by sex. The SEM analysis was conducted using Maximum Likelihood (ML) estimation. Given the large sample size (*n* = 2854), ML estimation was considered appropriate as it is robust to deviations from normality, even when variables are not normally distributed [83]. Although cybervictimization showed substantial deviations from normality, alternative robust estimators such as MLR or WLSMV could also be considered in such cases; however, ML was retained in the present study given the large sample size and its widespread use under these conditions. Importantly, the substantive pattern of results and the conclusions drawn from the models are based on standardized estimates and incremental fit indices, which are less sensitive to violations of multivariate normality than chi-square statistics. Model fit was assessed using recommended indices [84]: Chi-square (χ^2^), root mean square error of approximation (RMSEA), comparative fit index (CFI), Tucker-Lewis index (TLI), and standardized root mean square residual (SRMR). The model was considered to fit the data adequately if RMSEA ≤ 0.08, CFI and TLI ≥ 0.90, and SRMR ≤ 0.08. Multigroup invariance was assessed using three incremental levels: configural invariance, metric invariance, and scalar invariance. The models were compared through the difference in the values of CFI (ΔCFI) and RMSEA (ΔRMSEA). The values of ΔCFI < 0.01 and ΔRMSEA < 0.015 allow us to establish the invariance of the scale [85, 86].

## Results

### Descriptive statistics

The correlation analysis (see Table 1) showed significant associations between the resilience total and its dimensions, maladaptive schemas, victimization, and cybervictimization. However, spirituality did not show significant correlations with impaired autonomy or with the self-efficacy-tenacity dimension. Overall, the variables showed good reliability. However, the spirituality dimension presented lower internal consistency, likely due to the small number of items. The skewness and kurtosis values for normally distributed data were typically below ± 3 and ± 10, respectively, for all variables except cybervictimization [87]. The cybervictimization variable exhibited high skewness (4.16) and kurtosis (30.32), indicating a highly skewed distribution with heavy tails.

**Table 1:** Correlation matrix, descriptive statistics, and reliabilities for variables used in SEM

	**(1)**	**(2)**	**(3)**	**(4)**	**(5)**	**(6)**	**(7)**	**(8)**	**(9)**	**(10)**
Resilience Total (1)										
Rejection (2)	−0.30**									
Autonomy (3)	−0.30**	0.75**								
Victimization (4)	−0.17**	0.32**	0.30**							
Cybervictimization (5)	−0.14**	0.28**	0.27**	0.64**						
SET (6)	0.87**	−0.27**	−0.30**	−0.16**	−0.13**					
CUP (7)	0.90**	−0.20**	−0.21**	−0.12**	−0.08**	−0.21**				
ASN (8)	0.86**	−0.28**	−0.27**	−0.19**	−0.15**	−0.27**	0.74**			
CP (9)	0.74**	−0.33**	−0.32**	−0.20**	−0.15**	−0.32**	0.68**	0.71**		
S (10)	0.46**	−0.05**	−0.02	−0.05**	−0.04*	−0.02	0.47**	0.46**	0.44**	
Mean	25.56	44.54	21.84	3.99	1.38	21.76	17.19	13.41	7.90	4.54
SD	9.11	20.76	11.19	4.04	2.61	7.61	6.20	4.66	3.24	2.12
Skew	−0.58	0.95	0.98	1.74	4.16	−0.79	−0.46	−0.72	−0.66	−0.22
Kurto	−0.04	0.49	0.36	4.17	30.32	0.22	−0.03	0.07	−0.31	−0.56

Key: *Skew* Skewness, *Kurto* Kurtosis, *SET* Self-Efficacy-Tenacity, *CUP* Control under pressure, *ASN* Adaptability and support networks, *CP* A sense of control and purpose, *S* Spirituality

*p ≤.05

**p ≤.01

### Structural equation modelling

**Fig. 1:**
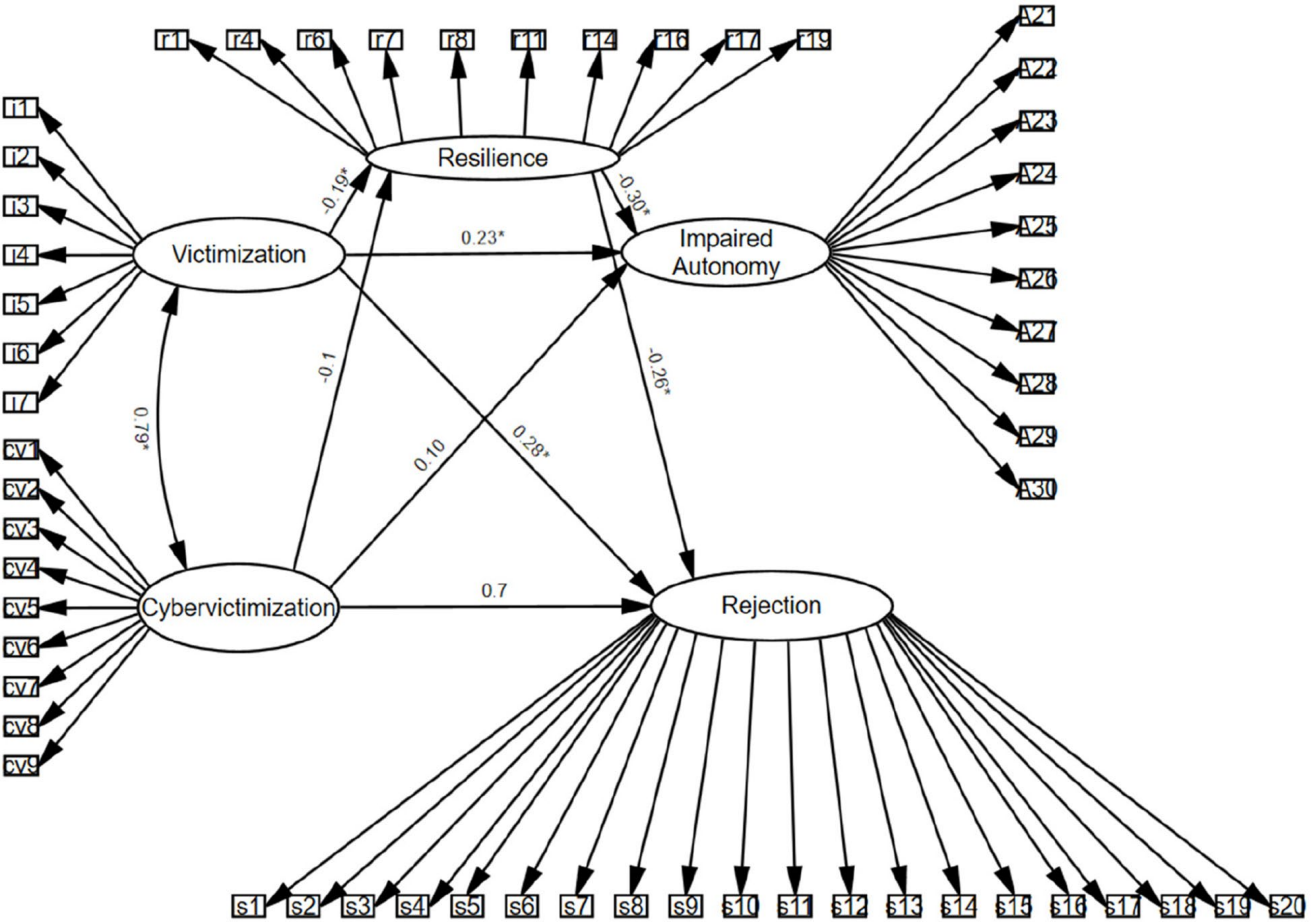
SEM of the mediating role of resilience in victimization, cybervictimization, impaired autonomy, and rejection schemas. Note: Standardized coefficients are presented. *p <.001

Before proceeding with the SEM (see Fig. 1), CFA was conducted to evaluate the measurement model. The results indicated a good fit to the data, with recommended fit indices achieved. Factor loadings were generally above 0.40, except for Item 9 of the Cybervictimization Scale, which had a factor loading of 0.34. Despite this exception, the measurement model demonstrated sufficient validity, providing a solid foundation for subsequent SEM analyses.

Table 2 summarizes the mediating role of total resilience and its specific dimensions (SET: self-efficacy-tenacity, CUP: control under pressure, ASN: adaptability and support networks, CP: control and purpose, and S: Spirutuality) in the relationship between victimization/cybervictimization and the maladaptive schemas of rejection and impaired autonomy.

**Table 2:** Mediation models of victimization and cybervictimization on maladaptive schemas through total resilience and its dimensions

Model	Mediator	Effect	Mediation Coefficient	Bootstrapping		Model Fit Indices (CFI, RMSEA, TLI, SMR)
				BootLLCI	BootULCI	
M1: Total	Resilience (10 items) Total Resilience	Victimization-Rejection	β = 0.048	0.025	0.075	CFI = 0.921, RMSEA = 0.038, TLI = 0.916, SRMR = 0.042
		Victimization-Autonomy	β = 0.055	0.028	0.084	
		Cybervictimization-Rejection	β = 0.002	−0.024	0.027	
		Cybervictimization-Autonomy	β = 0.002	−0.030	0.030	
M2: SET	SET	Victimization-Rejection	β = 0.299	0.013	0.057	CFI = 0.917 RMSEA = 0.041, TLI = 0.911, SRMR = 0.045
		Victimization-Autonomy	β = 0.240	0.016	0.073	
		Cybervictimization-Rejection	β = 0.067	−0.014	0.030	
		Cybervictimization-Autonomy	β = 0.090	−0.018	0.037	
M3: CUP	CUP	Victimization-Rejection	β = 0.302	0.012	0.053	CFI = 0.915, RMSEA = 0.040, TLI = 0.909, SRMR = 0.045
		Victimization-Autonomy	β = 0.247	0.014	0.062	
		Cybervictimization-Rejection	β = 0.079	−0.024	0.015	
		Cybervictimization-Autonomy	β = 0.104	−0.028	0.017	
M4: ASN	ASN	Victimization-Rejection	β = 0.281	0.026	0.081	CFI = 0.918, RMSEA = 0.041, TLI = 0.911, SRMR = 0.042
		Victimization-Autonomy	β = 0.229	0.027	0.086	
		Cybervictimization-Rejection	β = 0.064	−0.017	0.039	
		Cybervictimization-Autonomy	β = 0.088	−0.018	0.042	
M5: CP	CP	Victimization-Rejection	β = 0.245	0.053	0.126	CFI = 0.918, RMSEA = 0.042, TLI = 0.911, SRMR = 0.044
		Victimization-Autonomy	β = 0.188	0.058	0.136	
		Cybervictimization-Rejection	β = 0.083	−0.047	0.024	
		Cybervictimization-Autonomy	β = 0.108	−0.050	0.027	
M6: S	S	Victimization-Rejection	β = 0.317	−0.051	0.000	CFI = 0.915, RMSEA = 0.043, TLI = 0.908, SRMR = 0.044
		Victimization-Autonomy	β = 0.273	−0.040	0.000	
		Cybervictimization-Rejection	β = 0.083	0.000	0.053	
		Cybervictimization-Autonomy	β = 0.105	0.000	0.040	

Key: *SET* Self-efficacy-tenacity, *CUP* Control under pressure, *ASN* Adaptability and support networks, *CP* A sense of control and purpose, *S* Spirituality

Across all models, victimization showed stronger indirect effects through resilience dimensions compared to cybervictimization, particularly for rejection. Additionally, in the relationship between victimization and the maladaptive schemas, the resilience total, and the SET, CUP, ASN, and CP dimensions consistently demonstrated significant mediation effects. In contrast, the mediation effects of cybervictimization were weaker and mostly nonsignificant, as reflected in the low coefficients. However, a significant mediation effect of cybervictimization was observed in the “Spirituality” (S) dimension.

Model fit indices (CFI, RMSEA, SRMR) indicated good model performance, with values consistently meeting acceptable thresholds across all models. See Table 2 for detailed coefficients and model fit indices.

### Measurement invariance by sex

Table 3 presents the goodness-of-fit indexes of the mediation model with total resilience as the mediating variable for each of the groups by sex. In addition, it presents the measurement invariance models for these groups. The goodness-of-fit indexes showed adequate values, suggesting that the model is relevant for the subgroups. Concerning measurement invariance, as shown in Table 3, when comparing the restrictive models with the less restrictive ones, the values of ΔCFI < 0.01 and ΔRMSEA < 0.015 allowed us to establish the measurement invariance by sex in the three levels (configural, metric, and scalar). However, although ΔCFI = −0.013 exceeded the cutoff point, considering that ΔRMSEA = 0.002 is within the acceptable range, invariance was maintained. Additionally, regarding measurement invariance by sex, although the χ^2^ difference test was significant, changes in incremental fit indices were considered, given the large sample size. Since ΔCFI was close to the recommended cutoff and ΔRMSEA remained below 0.015, scalar invariance was supported.

**Table 3:** Confirmatory factor analysis and invariance tests for sex

Model	χ^2^	df	RMSEA (IC 90%)	CFI	SRMR	TLI	ΔM	Δχ^2^	Δdf	ΔRMSEA	ΔCFI
Sex											
1. Males N = 1347	4073.7*	1444	.037 (.035-.038)	.928	.041	.923	–	–	–	–	–
2. Females N = 1447	4639.9*	1444	.039 (.038-.040)	.908	.043	.902	–	–	–	–	–
M1	8713.5*	2888	.027 (.026-.028)	.918	.047	.913	–	–	–	–	–
M2	9499.5*	2939	.028 (.028-.029)	.908	.050	.904	M2 vs. M1	753.0*	51	0.001	−0.01
M3	10,516.15*	2995	.030 (.029-.031)	.895	.054	.892	M3 vs. M2	1016.6*	56	0.002	−0.013

*χ*^2^ Chi-square, *df* Degrees of freedom, *RMSEA* Root mean square error of approximation, *CFI* Comparative fit index, *SRMR* Standardized root mean square residual, *TLI * Tucker-Lewis index, *ΔΧ*^2^ Chi-square difference, *Δdf* Difference in degrees of freedom, *ΔRMSEA* Difference in root mean square error of approximation, *ΔCFI* Difference in comparative fit index, *M1* Configural model, *M2* Metric invariance, *M3* Scalar invariance

*p <.001

## Discussion

The present study provides evidence on the mediating role of resilience between victimization/cybervictimization and maladaptive schemas of disconnection/rejection and impaired autonomy. Thus, resilience acts as a variable of interest to help explain the dynamics of victimization/cybervictimization and their possible relationship with the internal universe of the victims through maladaptive schemas. Our SEM analyses showed that peer victimization, rather than cybervictimization, displayed the strongest direct and indirect associations with maladaptive schemas, and that resilience—especially self-efficacy/tenacity and control under pressure—partially mediated these links. These empirical patterns provide the basis for the interpretative arguments that follow.

On the one hand, it is expected that victimization/cybervictimization, as a potentially traumatic experience for the victims, is associated with more maladaptive cognitive schemas. Specifically, as previous evidence [25, 35, 36, 40, 41, 47] has already pointed out, the disconnection/rejection domain is particularly relevant in the context of victimization [40]. This dimension includes schemas that imply the expectation that a person's needs for security, acceptance, and respect will not be met predictably [37]. Therefore, it is coherent to expect that, after an experience with high traumatic content, such as victimization, feelings of distrust, abandonment, emotional deprivation, and deficiency may emerge in the victim [41]. Thus, adolescents who are rejected by their peers and experience insults and humiliation can develop cognitions and emotions such as feeling defective and some hostility, which is linked to the belief that others will intentionally harm them and, in turn, influences the victims' behavior [35, 36]. That is, an adolescent with a high rejection belief can make greater hostile attributions to the behavior of others in ambiguous contexts, leading the adolescent to react aggressively and thereby generating greater rejection [51, 88].

Focusing on impaired autonomy schemas, for the study sample, there was a significant relationship with victimization/cybervictimization through the direct effects of these variables. The results found are contrary to the bulk of previous studies that have considered that this dimension is less influential in victimization [35, 36, 41], although they are consistent with other studies that have demonstrated its relationship in situations of violence [51]. The dissonance of results may be due to the different instruments used to assess bullying behaviors, the type of behavior (verbal, social, physical, psychological, etc.), or form (direct or indirect) measured, as well as the stage of adolescence [23, 24]. In any case, it makes sense to expect that being a victim of (cyber)bullying is associated with higher leves of schemas associated with this domain. On the one hand, victims' fear of going through the same thing again can increase their feelings of vulnerability to harm [89, 90]. Likewise, being the object of bullying or cyberbullying may be associated with an irrational fear that something bad will happen, either through anticipatory anxiety [91, 92] or agoraphobia [93, 94]. Finally, failure is another of the schemas associated with this domain. The attack suffered by the victims, damaging their self-esteem [95] and making them feel unprotected from their aggressor or aggressors and the spectators that may accompany them as witnesses, can activate internal psychological mechanisms such as failure or fear of failure, which, in addition, significantly affect the protagonist's subjective well-being [96].

Once the relationship between (cyber)victimization and maladaptive schemas has been discussed, it is necessary to answer the first research question posed. In this regard, it should be noted that, in general, resilience acted as a partial mediating variable. Regarding traditional victimization, resilience partially mediated both as a global construct and considering all its dimensions (with the exception of "Spirituality").These results are consistent with previous studies in which the positive role of resilience in the dynamics of victimization has been observed [58–61, 69], showing it to be a great ally as a protective factor for victims [56, 62, 64, 72]. In other words, resilience or resistance in the face of adversity acts as a buffer against the harmful effects of peer violence in adolescents. Specifically, for the study sample, the feelings of self-efficacy, maintaining some control at times under pressure, having support networks and purposes help victimization generate fewer cognitive patterns of rejection and impaired autonomy. Specifically, these dimensions suggest that adolescents who believe in their ability to handle difficulties (self-efficacy) and can maintain calm in stressful situations (control under pressure) are better equipped to buffer the internalization of peer aggression. In practical terms, these coping capacities may prevent external victimization experiences from consolidating into internal beliefs of defectiveness or vulnerability, therefore interrupting the pathway to maladaptive schemas.

In the case of cybervictimization, however, resilience acted as a partial mediator only through the dimension of Spirituality. However, given the measurement limitations, this finding should be interpreted as exploratory This result is consistent with other authors who have highlighted the role of spirituality/religiosity as a strong coping strategy in traumatic situations [97, 98] such as cyberbullying [69, 99–101]. More specifically, our models indicated that only the spirituality dimension of resilience significantly mediated the association between cybervictimization and both maladaptive schemas, whereas the remaining resilience dimensions did not show meaningful indirect effects for cybervictimization. This specific pattern helps to contextualize the distinctive role of spirituality in online contexts. It is striking that, for the study sample, the spiritual dimension was a mediator only for cyberbullying, suggesting that the online universe gives bullying a unique entity that differentiates it from traditional bullying. In this case, it is to be expected that the RICT characteristics, such as 24 × 7 accessibility, the anonymity behind which the aggressor hides, the ubiquity, etc., may lead victims to deploy different meaning-based and intrapersonal coping strategies, such as spirituality [6]. In this sense, the spirituality dimension assessed in the CD-RISC reflects an internal search for meaning and transcendence rather than contextual or interpersonal coping resources, which may be particularly activated in cybervictimization contexts characterized by ambiguity and reduced external control. On the contrary, in traditional victimization, coping could be more oriented towards external resources, such as social support or the intervention of authority figures, which could explain the absence of a significant mediating effect of spirituality in this context. Starting from the premise that online reality has a unique nature that differentiates it from offline contexts (face-to-face), part of this dissonance may lie in the fact that these processes are not being evaluated by the same means. In other words, as resilience processes are corseted to the offline reality (traditional relational), resilience could likely have a unique nature in online realities. Based on this idea, it is possible that the instrument used (CD-RISC) could not capture this co-construction of the offline-online reality that is increasingly present in Generation Z and Generation Alpha [10]. This has occurred with other constructs such as Fear of Missing Out (FoMo) [102–104] or online emotional intelligence [105]. In any case, as previous authors have pointed out, spirituality can be a coping skill for adolescent resilience that can be a great ally in helping them recover from feelings of depression, anger, irritability, hopelessness, or anxiety [99, 101, 106], typical of situations of victimization.

Regarding the second research question, the findings show that the mediation model is invariant for boys and girls. This implies that the measurement structure is the same, which allows valid comparisons between the two groups of the relationships and levels (means) of the construct studied. Beyond the psychometric implications, the invariance of the model by sex suggests that the psychological mechanisms linking victimization and cybervictimization to maladaptive schemas through resilience operate similarly in boys and girls. Despite documented sex differences in the prevalence and expression of victimization experiences, the present findings indicate that resilience plays a comparable protective role across sexes in buffering the cognitive impact of peer violence [107, 108]. Nevertheless, these findings should be interpreted within the scope of the variables and measurement instruments included in the present study, and future research using longitudinal designs could further examine potential developmental or contextual variations in these mechanisms.

These results align with previous findings that suggest that stereotypes in anti-bullying and cyberbullying interventions must be overcome because interventions focused on psychological resilience will be equally effective for girls and boys, regardless of the role or behavior performed in bullying situations [56, 70, 71].

Given that resilience as a global construct and most of its dimensions partially mediated the link between traditional victimization and maladaptive schemas, and that spirituality partially mediated the association between cybervictimization and schemas, the practical implications of these findings underscore the importance of incorporating resilience-building components when designing psychoeducational prevention and intervention programmes. Identifying resilience as a mediating factor suggests that programmes should focus on strengthening adolescents’ ability to cope with victimization-related adversity. At the same time, it is crucial to intervene early to reduce the negative effects of victimization, offering strategies to prevent the formation of maladaptive schemas that can affect mental health in the long term. These programs must be flexible and personalized, adapting to individual characteristics and promoting both emotional well-being and the development of a more positive view of themselves and the environment. In addition, within the tutorial action programs carried out by the school guidance team, specific sessions could at least be incorporated to address resilience in the school context. In light of the results, one could consider the need to create specific resilience strategies for online contexts to aid with these phenomena, as well as to adapt the questionnaires to the online nature of the phenomenon.

This study presents some limitations. First, only self-report measures were used, which can lead to response bias and social desirability bias. Second, there may be a retrospective recall bias, as participants were asked to recall victimizing behaviors they had received in the past 4 months. Third, although the sample of participants was large and geographically dispersed, sampling was not random, so it is not representative of the Spanish context. Fourthly, although all the dimensions used present adequate reliability indicators, the dimension of spirituality, as part of resilience, showed results below 0.60. This result is likely attributable to the two-item format of this subscale, which is known to negatively affect reliability coefficients such as Cronbach’s alpha. Importantly, brief measures, including two-item scales, can still demonstrate adequate validity and theoretical relevance, particularly when assessing conceptually narrow constructs. Nevertheless, lower reliability may reduce measurement precision and attenuate effect sizes in structural models. Accordingly, mediation effects involving spirituality should be interpreted with caution, not because the construct lacks relevance, but because the estimates may be more sensitive to random measurement error. Future research would benefit from using extended or alternative measures of spirituality to increase precision and confirm the robustness of these findings. Fifth, all the variables used, except cybervictimization, have normal distributions. This pattern matches the phenomenon's nature, with most participants reporting little or no cybervictimization, while a minority reported exceptionally high levels of cybervictimization. Despite this, the CFA and SEM analyses were performed using ML estimation, as the large sample size (*n* = 2854) helps mitigate possible biases due to non-normality. The ML estimate is robust to deviations from normality when working with large datasets [83]. Finally, some model fit indices are slightly below the thresholds considered good (in particular, CFI and TLI), but all of them can be considered acceptable. However, the overall pattern of fit is supported by adequate RMSEA and SRMR values, as well as the theoretical coherence and complexity of the model, which allows the model to be considered acceptable despite these marginal incremental indices.

To conclude, the results suggest the mediating role, both of resilience as a global construct and of all its dimensions except for Spirituality between traditional victimization and the maladaptive schemas of disconnection and rejection and impaired autonomy. On the contrary, only the Spirituality dimension partially mediated between cybervictimization and both maladaptive schemas, a finding that requires cautious interpretation and further analysis and replication.

## Data Availability

The data that support the findings are available from the authors upon request.
